# A protective multiple gene-deleted African swine fever virus genotype II, Georgia 2007/1, expressing a modified non-haemadsorbing CD2v protein

**DOI:** 10.1080/22221751.2023.2265661

**Published:** 2023-10-19

**Authors:** Anusyah Rathakrishnan, Ana L. Reis, Vlad Petrovan, Lynnette C. Goatley, Katy Moffat, Yuan Lui, Mai T. Vuong, Shinji Ikemizu, Simon J. Davis, Linda K. Dixon

**Affiliations:** a The Pirbright Institute, Woking, UK; b Radcliffe Department of Medicine and Medical Research Council Human Immunology Unit, John Radcliffe Hospital, University of Oxford, Oxford, UK; c Graduate School of Pharmaceutical Sciences, Kumamoto University, Kumamoto, Japan

**Keywords:** African swine fever, Virulence, Modified live vaccine, CD2v, Haemadsorption

## Abstract

African swine fever virus is a complex DNA virus that causes high fatality in pigs and wild boar and has a great socio-economic impact. An attenuated genotype II strain was constructed by replacing the gene for wildtype CD2v protein with versions in which single or double amino acid substitutions were introduced to reduce or abrogate the binding to red blood cells and reduce virus persistence in blood. The mutant CD2v proteins were expressed at similar levels to the wildtype protein on the surface of infected cells. Three recombinant viruses also had K145R, EP153R, and in one virus DP148R genes deleted. Following immunization of pigs, the virus with a single amino acid substitution in CD2v, Q96R, induced moderate levels of replication, and 100% protection against virulent ASFV. Two additional recombinant viruses had two amino acid substitutions in CD2v, Q96R, and K108D, and induced no binding to red blood cells *in vitro*. In immunized pigs, reduced levels of virus in blood and strong early ASFV-specific antibody and cellular responses were detected. After challenge low to moderate replication of challenge virus was observed. Reduced clinical signs post-challenge were observed in pigs immunized with the virus from which DP148R gene was deleted. Protection levels of 83–100% were maintained across a range of doses. Further experiments with virus GeorgiaΔDP148RΔK145RΔEP153R-CD2v_mutantQ96R/K108D showed low levels of virus dissemination in tissue and transient clinical signs at high doses. The results support further evaluation of GeorgiaΔDP148RΔK145RΔEP153R-CD2v_mutantQ96R/K108D as a vaccine candidate.

## GRAPHICAL ABSTRACT

**Figure:**
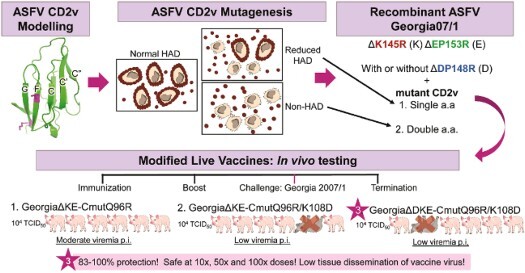

## Introduction

African swine fever virus (ASFV) can cause death of most infected pigs and has a high socio-economic impact. Since the introduction from Africa to Georgia in 2007, ASF has spread extensively in Europe, Asia, and the Caribbean (FAO, WOAH). Deleting some genes attenuates disease in pigs and induces variable levels of protection against challenge [1–3]. We deleted or modified four genes in the genotype II Georgia07/1 virus and tested three different recombinant viruses for their attenuation and induction of protection against challenge in pigs. A key step was to modify the EP402R gene such that the encoded CD2v transmembrane protein lacks the undesired function of binding to red blood cells (RBC), haemadsorption (HAD), while maintaining cell surface expression [4–7]. The HAD phenotype facilitates virus spread and persistence in infected animals by maintaining a large proportion of ASFV in blood’s RBC fraction [6–8]. We showed previously that deleting the EP402R gene from a partially attenuated isolate, BeninΔDP148R [7,9], dramatically reduced the period of virus persistence in blood. Deletion of both EP402R and the C-type lectin EP153R genes, strongly attenuated virulent ASFV, but levels of protection induced following challenge were lower [7].

CD2v has immunomodulatory activities potentially affecting T cell activation [6] and the cytoplasmic domain can suppress type I interferon production by negatively regulating cGMP-AMP synthase-STING [10]. In infected cells, a C-terminal 26 kDa cleavage product is detected in addition to an N-terminal fragment and full-length protein [11].

Here, we replaced the wildtype EP402R gene with mutants containing single or double amino acid substitutions in the N-terminal (Ig1) domain. This reduced or abrogated the HAD phenotype but maintained cell surface expression of the protein. We also deleted K145R and EP153R genes alone or combined with DP148R gene [12]. Three novel recombinant ASFVs were tested by immunization and challenge in pigs. High levels of protection against challenge, between 83% and 100%, were achieved. One recombinant virus was attenuated even at high doses, up to 10^6^ HAD_50_. The results confirmed this combination of gene deletions and CD2v modification as a good approach to produce safe and efficacious modified live vaccine candidates with potential for application to different ASFV genotypes.

## Methods

### Cells and viruses

Vero cells were cultured in DMEM with 10% foetal bovine serum (FBS) and 1% penicillin–streptomycin (pen-strep). Porcine bone marrow cells (PBMs) were maintained in EBSS with 10% pig serum and 1% pen-strep [13]. ASFV strains were grown and titrated in PBMs [12,14].

### Haemadsorption (HAD) assay

Plasmids expressing wildtype or mutant CD2v were transfected into Vero cells (TransIT®-LT1, Mirus Bio) and incubated at 37°C for 48 h (h). RBC were added and after 16 h at 37°C, observed for the presence of rosettes. Expression of CD2v in Vero cells was confirmed using western blots and immunofluorescence (Supplementary Data).

### Production of ASFV modified Georgia07/1 with deletions of K145R, EP153R, DP148R, and expressing modified CD2v proteins

The genes deleted were EP153R (E), K145R (K), and DP148R (D) in one virus and EP402R was replaced with mutant versions by homologous recombination on the ASFV Georgia07/1 (FR682468.2) (Figure S1A). Parental viruses GeorgiaΔK145R or GeorgiaΔDP148RΔK145R expressing TagRFP-T were used as the backbone [12,15]. Recombinant (i) GΔKE_CmutQ96R codes for CD2v with a single Q to R aa substitution at residue 96; Recombinant (ii) GΔKE_CmutQ96R/K108D, has a double Q96 to R and K108 to D substitution in CD2v; Recombinant (iii) GΔDKE_CmutQ96R/K108D, has the same double amino acid substitution and additionally has DP148R deleted. Full genome sequences confirmed the expected deletions, reporter gene insertions, and mutations (Figure S1B). The GΔKE_CmutQ96R/K108D virus did not have the HA tag fused at the 3′ end of the wildtype sequence, indicating that the recombination occurred downstream of the mutated residues but upstream of the CD2v stop codon. Growth of recombinant viruses was compared to Georgia07/1 using multistep growth curves (Supplementary Data).

### In vivo immunization and challenge

**Figure 1.**
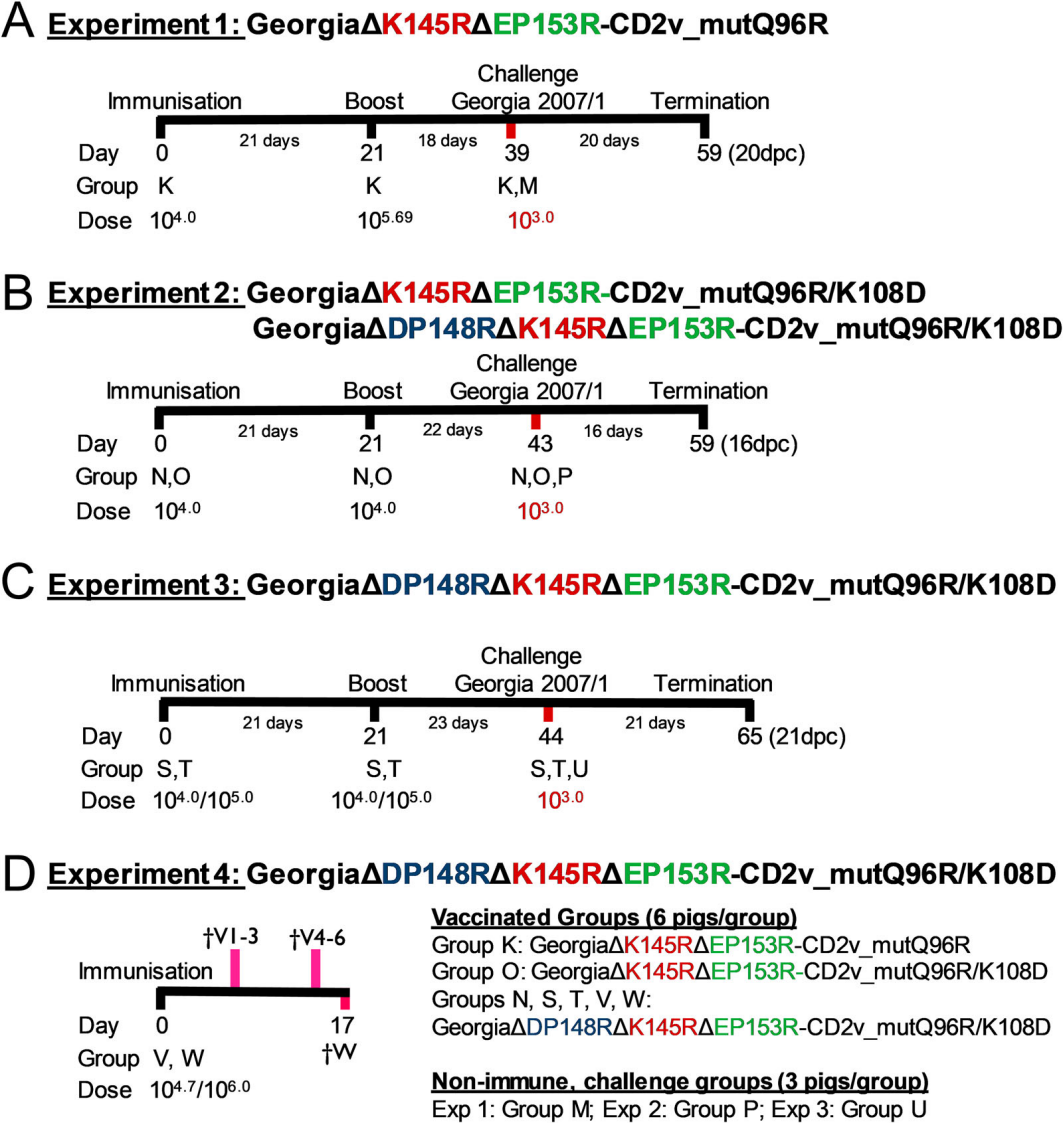
Experimental timeline. The timeline for four pig immunization experiments is shown. In experiment 4 shown in panel D † denotes the pigs and shows days that they were culled. In experiments 1, 2, and 3, groups of 3 non-immune pigs were challenged in parallel to the immunized pigs.

Groups of six female Landrace-Large White cross pigs with initial weights of 17–30 kg were inoculated and challenged via the intramuscular route (IM) (Figure 1). A group of three non-immune pigs were challenged in parallel as controls. Clinical signs of disease were recorded daily and blood samples were collected on selected days [16]. In experiment 1, one group (K) was immunized with GΔKE-CmutQ96R. In experiment 2, one group was immunized with GΔKE-CmutQ96R/K108D (O) and a second group with a virus also lacking the DP148R gene, GΔDKE-CmutQ96R/K108D (N). In experiment 3, two groups of pigs were immunized with different doses of GΔDKE-CmutQ96R/K108D. In experiment 4, two groups of pigs were immunized with two higher doses of GΔDKE-CmutQ96R/K108D and culled between 7 and 17 days later to investigate the tissue dissemination of the virus. Viruses were back titrated to verify dosages used. Pigs were euthanized at the termination of the experiment or at a moderate severity humane endpoint. Macroscopic lesions associated with ASFV were scored [17]. Viremia was evaluated by titration, while levels of genome copies in tissues were determined by qPCR. The levels of ASFV-specific antibodies were determined using a commercial kit (P72) or in-house ELISAs (CD2v and P30). IFN-γ cellular responses were measured as described previously [18]. Expanded methodologies are provided in the Supplementary Data.

### Statistical analyses

Statistical analyses were performed using GraphPad Prism 9.0.2 and is further discussed in the Supplementary Data.

## Results

### Transient expression of wildtype and mutant CD2v proteins to identify residues required for haemadsorption

**Figure 2.**
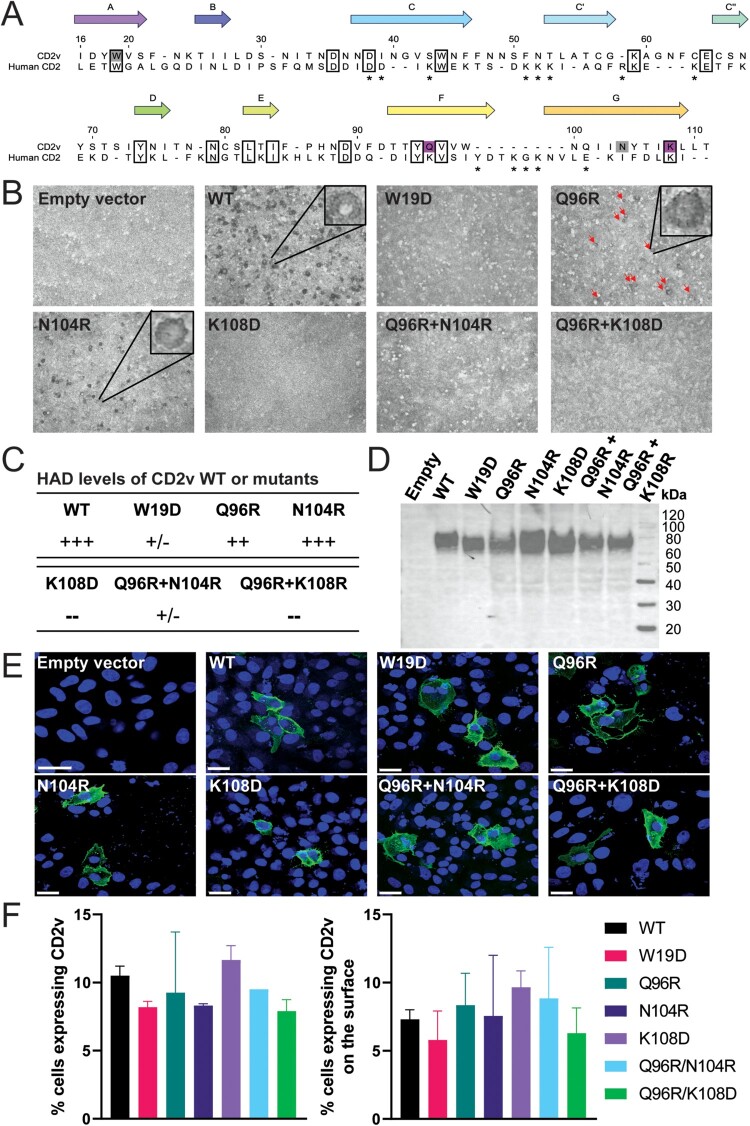
Sequences and transient expression of wildtype and mutant CD2v. Panel (A) shows the structure-based alignment of the putative CD2v and human CD2 Ig1 domains. Residues with 100% identity are boxed, and numbering is according to the CD2v sequence. Mutations that showed strong effects in the mutation screen are coloured magenta and weak grey. β-strands are labelled according to the human CD2 structure (PDB: 1HNF). Asterisks denote human CD2-CD58 interface residues (PDB: 1QA9). The accession numbers for these sequences are: CD2v Georgia, YP_009927182 (NCBI), and human CD2, P06729 (UniProt). For Panel (B), Vero cells were transfected with pcDNA3.1 plasmids expressing Georgia CD2v, wildtype (WT), or mutants with indicated single or double amino acid substitutions. After 48 h, RBC were added, or the cells were fixed and stained for expression of HA tag fused downstream of the signal peptide at the N-terminus of CD2v. Representative fields demonstrating the presence or absence of RBC rosettes (HAD), as indicated, are shown. Panel (C) summarizes results from repeat experiments and different fields representing HAD relative to wildtype CD2v: +++ similar levels; ++ reduced levels; +/− occasional detection of very low HAD not observed in a repeated experiment; and – absence of HAD. Panel (D) shows Western blots of lysates from cells transfected with plasmids expressing WT or mutant CD2v probed with anti-HA antibody. Molecular weight markers are indicated. Panel (E) shows WT or mutant CD2v detected on the surface of fixed, non-permeabilized cells (in green), probed with rat anti-HA, followed by goat anti-rat Alexa-Fluor 488 and counterstained with DAPI to show the nucleus (blue). The scale bars represent 25 µm. Additional images of permeabilized and non-permeabilized cells expressing CD2v at magnification of 20× and 63× are shown in Figure S3. The number of transfected cells expressing CD2v intracellularly or on the cell surface was quantified based on the 20× confocal images on Imaris software and the percentages are given in panel (F). No statistical differences were observed between CD2v WT and the mutants.

Previously (Reis et al., in preparation), we identified residues from the genotype I Benin97/1 CD2v Ig1 ligand-binding domain that were important for binding of CD2v to RBC, based on homology modelling with human CD2 [19]. AlphaFold2 (https://alphafold.ebi.ac.uk/) subsequently supported the overall correctness of the initial model generated by manual alignment (Reis et al., in preparation). Using the model, residues predicted to be on the ligand-binding GFCC'C″ β-sheet on genotype II Georgia07/1 CD2v protein were identified (Figures 2(A) and S2A), and “drastic” amino acid changes were made [20]. The CD2v wildtype gene, along with four single and two double-residue mutants were codon-optimized for mammalian expression with an in-frame HA epitope tag encoded at the N-terminus after the signal peptide. Plasmids encoding the genes were transfected into Vero cells and pig RBC were added to trypsin-treated cells. The formation of RBC rosettes by cells expressing mutant or wildtype CD2v was observed (Figure 2(B)) and qualitatively scored (Figure 2(C)). The W19D and K108D mutations dramatically reduced or completely abolished HAD, respectively, whereas the Q96R and N104R had smaller, or no effects on HAD. Mutating both Q96R and N104R or both Q96R and K108D, also abolished HAD. To ensure that the non-HAD phenotype was not due to reduced expression of the CD2v, near-equivalent total expression of the proteins was confirmed by Western blotting (Figure 2(D)) and similar numbers of transfected cells by immunofluorescence (Figure 2(F)). Transfected cells immunostained for HA without permeabilizing the cells also confirmed cell surface expression of CD2v (Figures 2(E) and S3).

### Characteristics of recombinant ASFV Georgia07/1 expressing mutated forms of CD2v

**Figure 3.**
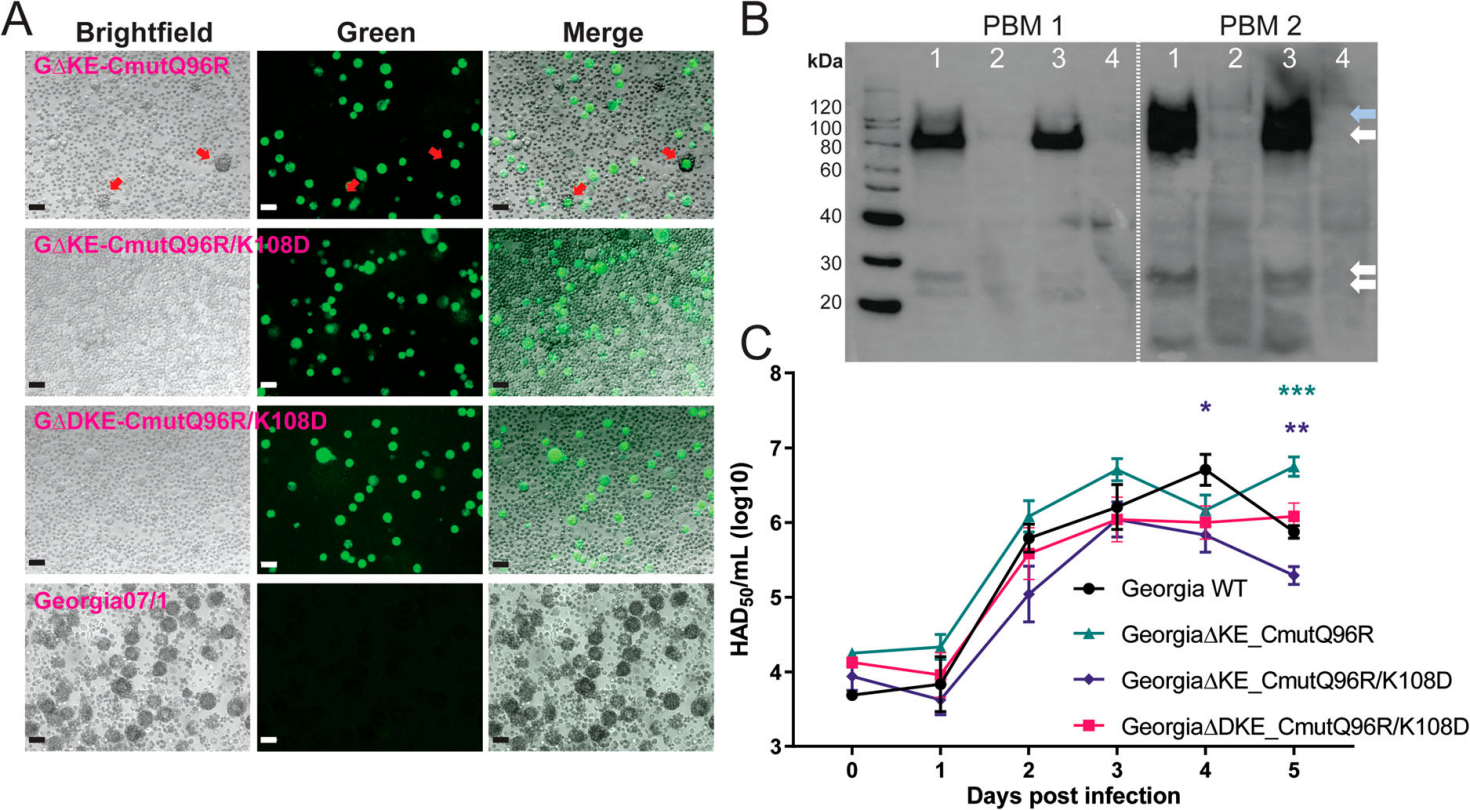
*In vitro* characterization of ASFV recombinant viruses expressing mutant CD2v. Panel (A) shows brightfield images of PBM cells infected with the recombinant or wildtype Georgia07/1 viruses, mNeonGreen expression in infected cells (green), and a merged image overlaying brightfield and green channels. The red arrows in GΔKE_CmutQ96R-infected cells show reduced numbers of rosettes compared to the wildtype virus. The scale bars represent 25 µm. Panel (B) shows Western blots of lysates of PBMs from two pigs infected at 0.1 MOI with GΔKE_CmutQ96R (Lane 1) and GΔDKE_CmutQ96R/K108D (Lane 3) at 4 dpi and probed to detect the C-terminal HA epitope tag. HA-specific bands were not detected in lanes with mock-infected (lane 4) or GΔKE_CmutQ96R/K108D-infected cell lysates (lane 2). This virus lacks the C-terminal HA tag. Arrows show bands of expected molecular weight for full-length glycosylated (∼80 kDa) CD2v or the C-terminal cleavage fragment. The blue arrow shows a higher MW (∼120 kDa) CD2v detected at higher levels lysates from infected PBMs from pig 2. Panel (C) shows titres of virus harvested from combined cell extracts and supernatants at different days post-infection of PBMs infected with wildtype or recombinant ASFV at 0.01 MOI in triplicate. Significant differences are represented by asterisk where * is *p* < 0.05, ** is *p* < 0.01, *** is *p* < 0.001, and **** is *p* < 0.0001; the colour of the asterisk represents the respective virus.

We selected single residue mutation Q96R and double mutation Q96R/K108D to introduce into recombinant viruses. The Q96R mutation was selected since it was similar to the E99R non-HAD mutation we tested in genotype I (Reis et al., in preparation) and it was interesting to compare a partially HAD mutation with the Q96R/K108D non-HAD mutation. The double Q96R/K108D mutation was also selected to decrease the chance of reversion to HAD phenotype during passage in pigs. EP153R and K145R genes were deleted from all three viruses and DP148R in addition from GΔDKE_CmutQ96R/K108D. The HAD phenotypes of the wildtype Georgia07/1 and the three recombinant ASFVs expressing mutant CD2v were compared by infection of PBM cells (Figure 3(A)). Similar results were obtained as in transient expression experiments, GΔKE_CmutQ96R induced partial HAD, whereas recombinants with double-residue mutations Q96R and K108D, induced no detectable HAD (Figure 3(A)). The non-HAD phenotype of these two recombinants did not alter after five serial passages in PBMs. Western blotting against the C-terminal HA tag, included in the recombinant viruses instead of the N-terminal tag used in the transfected cells, detected bands of MW ∼80, 120, and 26 kDa in cell lysates from the GΔKE_CmutQ96R and GΔDKE_CmutQ96R/K108D-infected PBMs (Figure 3(B)). These correspond to the full length glycosylated CD2v and cleavage product [11]. No bands were observed in extracts from cells infected with GΔKE_CmutQ96R/K108D since this virus does not have the C-terminal HA tag fused to CD2v. Analysis of the replication kinetics of the three recombinant and wildtype viruses in PBMs revealed no significant differences from days 1 to 3 post-infection (Figure 3(C)) and similar peak levels of virus replication (10^6.04-6.75^ TCID_50_/mL).

### Immunization of pigs with three recombinant ASFV induced high levels of protection against challenge with virulent Georgia07/1

#### Experiment 1. GeorgiaΔK145RΔEP153R-CD2v_mutantQ96R

**Figure 4.**
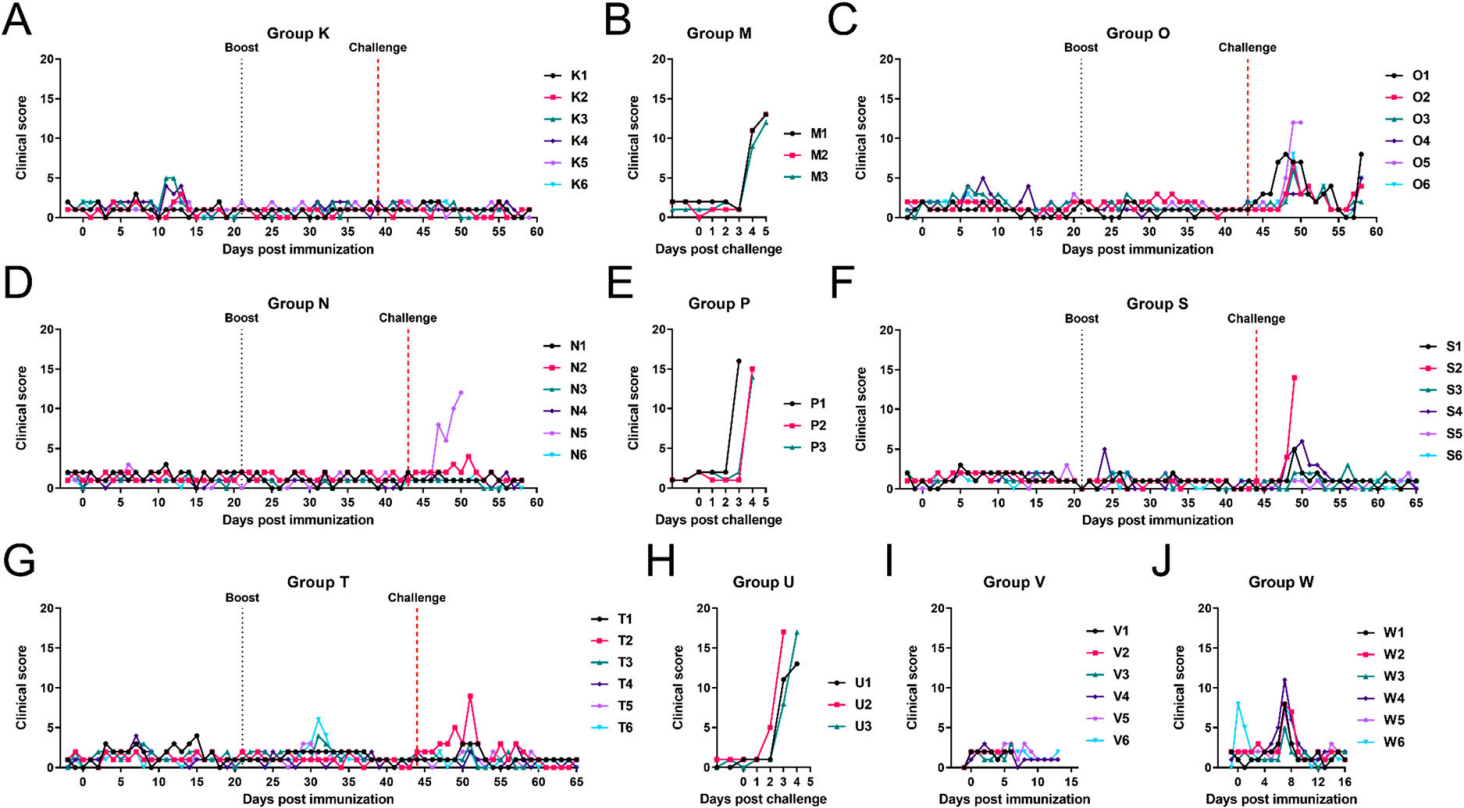
Clinical scores of pigs. Cumulative clinical scores per day for pigs immunized with (A) GΔKE_CmutQ96R (Group K), (C) GΔKE_CmutQ96R/K108D (Group O), and (D, F, G, I, and J) GΔDKE_CmutQ96R/K108D (Groups N, S, T, V, and W). (B, E, H) The cumulative scores per day for the naïve challenge control groups for each experiment were recorded following challenge with Georgia 2007/1 isolate.

**Figure 5.**
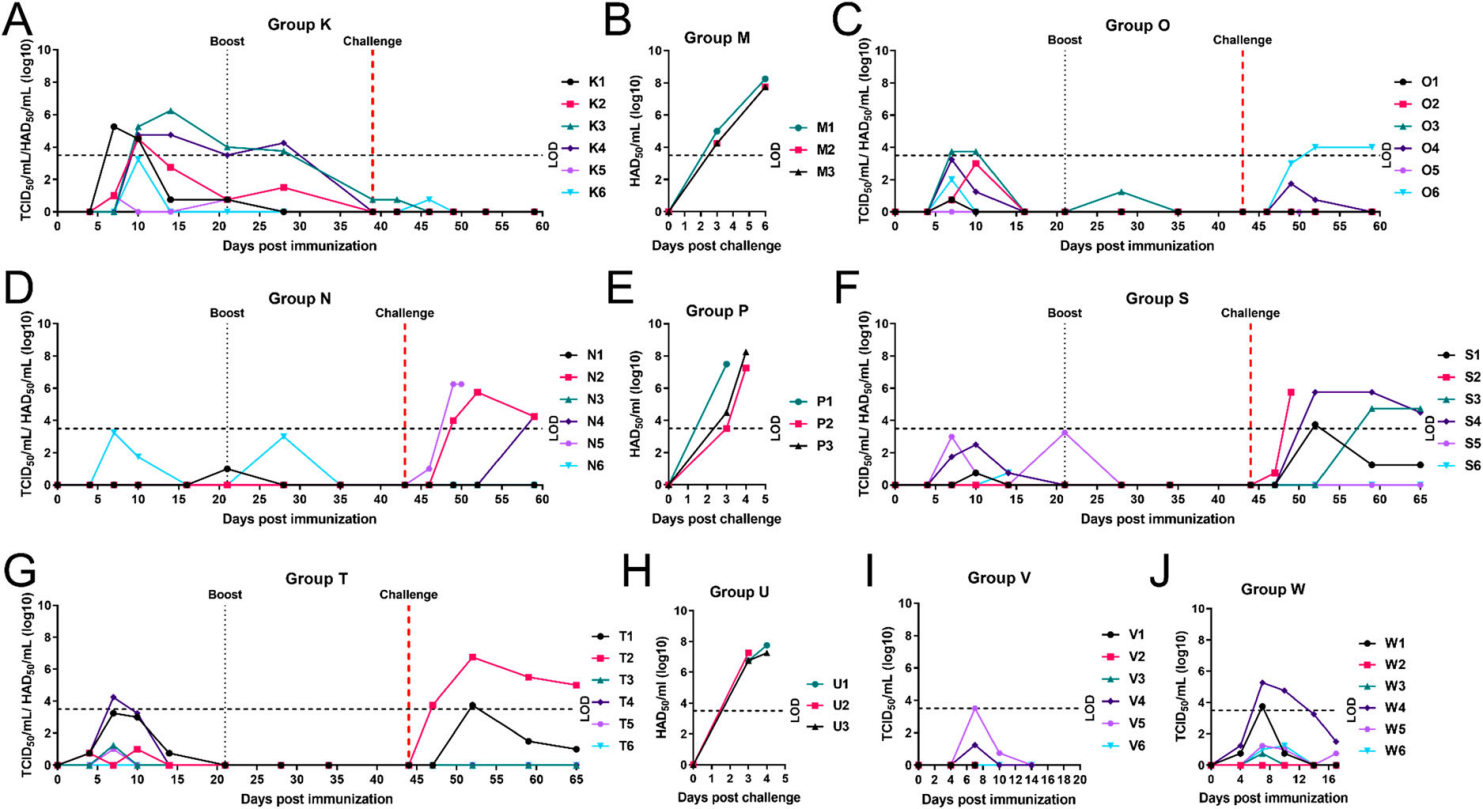
Level of viremia in pigs. Virus titres present in whole blood collected at different days post-immunization or challenge are shown for individual pigs as indicated. Pigs were immunized with (A) GΔKE_CmutQ96R (Group K), (C) GΔKE_CmutQ96R/K108D (Group O), and (D, F, G, I, and J) GΔDKE_CmutQ96R/K108D (Groups N, S, T, V, and W). Panels B, E, H show virus titres detected in blood from control non-immune pigs challenged with Georgia 2007/1 isolate. The dashed line on the *y*-axis represents the cut-off below which accurate measurement of infectious virus per mL blood was not obtained.

A summary showing results from experiments 1 to 4 is given in Table 1. Six pigs (Group K) were immunized and boosted on day 21 with 10^4^ TCID_50_ GΔKE_CmutQ96R in 1 mL PBS (Figure 1(A)). Two pigs developed temperatures above 40.5°C for 2 days but no other disease signs were observed (Figures 4(A) and S4A). Mild to moderate levels of viremia were detected in immunized pigs between 7 and 14 dpi (Figure 5(A)) peaking at 10^5.25-6.25^ TCID_50_/mL.

**Table 1.** Summary of *in vivo* results using the three different recombinant ASFV.

		Pre-challenge					Post-challenge				
		Dose^a^	Clinical signs		Viremia			Clinical signs		Viremia	
Recombinant ASFV	Group		>40.5°C	Signs^b^	# pigs	TCID_50_/mL	Protection^c^	>40.5°C	Signs	# pigs	HAD_50_/mL^d^
GΔKE_CmutQ96R	K	10^4^	2/6	0/6	6/6	10^0.75-6.25^^e^	100.0%	0/6	0/6	2/6	10^0.75^^f^
GΔKE_CmutQ96R/K108D	O	10^4^	2/6	0/6	5/6	10^0.75-3.75^	83.3%	1/6	6/6	2/6	10^1.75-4.00^
GΔDKE_CmutQ96R/K108D	N, S	10^4^	0/12	1/12	6/12	10^0.75-3.25^	83.3%	5/12	3/12	7/12	10^0.75-6.25^
GΔDKE_CmutQ96R/K108D	T	10^5^	1/6	1/6	5/6	10^0.75-4.25^	100%	1/6	5/6	2/6	10^1.00-6.75^
Georgia07/1^g^	M, P, U	–	–	–	–	–	0%	9/9	9/9	9/9	10^7.25-8.25^

^a^ Prime and boost dose is given as TCID_50_ and was administered intramuscularly in 1 ml PBS.

^b^ Signs refer to every other clinical signs observed daily besides temperatures >40.5°C.

^c^ Percentage of pigs that survived Georgia07/1 challenge.

^d^ Only challenge virus was detected post-challenge and the titres are expressed as HAD_50_/mL in all cases except for Group K.

^e^ Virus isolated from blood of Group K pigs was haemadsorbing.

^f^ Virus was isolated from 2 Group K pigs at low levels – one vaccine virus and the other challenge virus.

^g^ Non-immunized control pigs challenged at the same time as the immunized pigs.

After challenge, the three non-immune pigs (Group M) were euthanized at 6dpc showing typical acute ASFV signs (Figures 4(B) and S4B). The Group K pigs did not develop any signs after challenge and all survived (Figures 4(A) and S4A). ASFV was detected in blood below the limit of accurate measurement (LOD) in two pigs after challenge. A low level of the vaccine virus was isolated on 3dpc in one pig and low levels of challenge virus in another pig 7dpc (Figure 5(A)). The non-immune Group M had high levels of viremia, 10^7.75-8.25^ HAD_50_/mL, by 6dpc (Figure 5(B)). Necropsy of non-immune pigs showed gross lesions typical of acute ASF infection. The vaccinated pigs terminated at 20dpc had mild lesions, mostly enlargement of one or 2 lymph nodes, or mild ascites or hydropericardium (Figure S5A).

#### Experiment 2. GeorgiaΔK145RΔEP153R-CD2v_mutantQ96R/K108D compared to GeorgiaΔDP148RΔK145RΔEP153R-CD2v_mutantQ96R/K108D

In experiment 2, we compared two recombinant viruses expressing CD2v with double mutations of Q96R and K108D. These viruses differed since one virus had an additional deletion of DP148R gene (Figure 1(B)). The six pigs in Group O were immunized and boosted with 10^4^ TCID_50_ of GΔKE_CmutQ96R/K108D in 1 mL PBS, which has an intact DP148R gene. After immunization, two pigs had transient temperatures above 40.5°C for 1 day or for 3 days, on 6, 8, and 15 dpi (Figures 4(C) and S4C) but no other disease signs. Low viremia was detected in five pigs and none in the other pig after immunization (Figure 5(C)). Thus, the additional mutation in the CD2v protein, which completely abrogated HAD, resulted in lower viremia compared to Group K (GΔKE_CmutQ96/R).

After challenge, one pig was culled at the moderate severity endpoint on 7dpc. None of the other Group O pigs developed temperatures, although all had other mild to moderate disease signs on varying days until termination of the study (Figures 4(C) and S4C). No viremia was detected in four of the Group O pigs, including the pig that was culled at 7dpc. One pig had levels below the LOD and another had peak levels on 9 and 16dpc, at 10^4.0^ HAD_50_/mL (Figure 5(C)).

At necropsy, all Group O pigs showed mild macroscopic lesions such as enlargement of lymph nodes, tonsils, or spleen, with slight haemorrhages and mild lung pathology. Additionally, one pig also had clear, gelatinous ascites, enlarged liver, and fibrinous pericarditis (Figure S5B).

The second group of six pigs (Group N) in experiment 2 was immunized and boosted with 10^4.0^ TCID_50_ of GΔDKE_CmutQ96R/K108D in 1 mL PBS, which had an additional deletion of DP148R gene. No clinical signs were observed in this group after immunization (Figures 4(D) and S4D). Low levels of viremia below the LOD were detected in two pigs (Figure 5(D)).

After challenge, one pig was culled at the humane endpoint on day 7pc. A second pig had elevated temperature 8dpc but showed no other clinical signs. No other pigs in Group N displayed clinical signs until the end of the study (Figures 4(D) and S4D). No viremia was detected in three pigs after challenge (Figure 5(D)). The pig culled at the moderate endpoint, had 10^6.25^ HAD_50_/mL on 7dpc. One pig had peak viremia on 9dpc at 10^5.75^ HAD_50_/mL; and another only had viremia at the end of the study at 10^4.25^ HAD_50_/mL.

Group N pigs had mild macroscopic scores which included occasionally splenomegaly, lymph node enlargement, and/or the presence of hydropericardium. One pig had ascites and partial haemorrhagic tracheobronchial lymph nodes. Four pigs showed evidence of mild lung oedema (Figure S5B).

Non-immune pigs in control Group P, all developed acute ASFV signs and were culled on 3–4dpc at the moderate severity endpoint (Figures 4(E) and S4E). All had high levels of virus in blood (Figure 5(E)) and macroscopic lesions typical of acute ASFV (Figure S5B).

Recombinant virus GΔDKE_CmutQ96R/K108D showed lower clinical signs post-challenge and thus an improved safety profile supporting its further evaluation.

#### Experiment 3: higher dose (10×) immunization with ASFV GΔDKE_CmutQ96R/K108D

In experiment 3, two groups of six pigs (Groups S and T respectively) were immunized and boosted at doses of 10^4.0^ and 10^5.0^ TCID_50_, in 1 mL PBS with GΔDKE_CmutQ96R/K108D (Figure 1(C)). Group S pigs exhibited no clinical signs after immunization or boost (Figures 4(F) and S4F). Low levels of viremia below the LOD were detected in four of the Group S pigs (Figure 5(F)). These results were comparable to those obtained for Group N, which were immunized with the same dose of this virus. Group T pigs were immunized with a 10× higher dose, and four developed transient temperatures >40.5°C (Figures 4(G) and S4G). Viremia below the LOD was detected in four pigs, and pig T6 had no detectable viremia. Pig T4 had peak levels on 7 dpi, at 10^4.25^ TCID_50/_mL (Figure 5(G)).

Five of six pigs in Group S survived challenge showing transient mild or moderate signs (Figure 4(F)). Two pigs had no viremia after challenge, and one pig had low levels peaking at 8dpc with 10^3.75^ HAD_50_/mL (Figure 5(F)). The remaining three pigs had moderate levels of viremia including the pig which was culled at the humane endpoint. This pig had 10^5.75^ HAD_50_/mL at termination.

All Group T pigs survived challenge. Only one pig had an elevated temperature >40.5°C and reduced eating on two days (Figure 4(G)). Four pigs had no viremia after challenge and one pig had low levels of viremia peaking at 8dpc with 10^3.75^ HAD_50_/mL (Figure 5(G)). Pig T2 had moderate levels of viremia, at 8dpc (10^6.75^ HAD_50_/mL), declining over time. The non-immune Group U pigs presented with typical acute ASFV signs and were culled at the humane endpoint on 3 or 4dpc (Figures 4(H) and S4H). Non-immune pigs had viremia levels of >10^7.25^ HAD_50_/mL at termination (Figure 5(H)).

Virus quantities in six different tissues from Groups S, T, and U after challenge with Georgia07/1 showed that the control Group U pigs had moderate to high levels of ASFV genome in all six tissues (Table S1). In contrast, Group S surviving pigs had low levels of ASFV genome copies ranging from 10^0.69^ genome copies/g in the submandibular lymph node to 10^4.32^ copies/g in the gastrohepatic lymph node. Four pigs in Group T had low ASFV genome copies in tissues at maximum 10^3.92^ genome copies/g in tonsils of one pig (Table S1). Infectious virus was not isolated from any tissues of pigs from Groups S and T.

### Further safety testing of GeorgiaΔDP148RΔK145RΔEP153R-CD2v_mutantQ96R/K108D

#### Experiment 4: short-term immunization study showing tissue dissemination of GeorgiaΔDKE-CmutQ96R/K108D

In experiment 4, two groups of six pigs were immunized IM with 1 mL of GeorgiaΔDKE-CmutQ96R/K108D at 10^4.7^ TCID_50_ (Group V) or 10^6.0^ TCID_50_ (Group W). Tissues were collected to measure virus dissemination from 3 pigs in Group V on 7 dpi and 3 on 14 dpi (Figure 1(D)).

None of the Group V pigs displayed clinical signs after immunization. Viremia was detected in two pigs at or below the LOD on 7 dpi (Figures 4(I), 5(I), and S4I). Pigs in Group W were given 100× dose compared to Groups N and S and were terminated on 17 dpi. All had reduced eating on 7 dpi (Figure 4(J)). Two pigs had transiently increased temperatures above 40.5°C (Figure S4J). All six pigs recovered and showed no further clinical signs until termination. Viremia was below the LOD in three pigs and not detected in one pig (Figure 5(J)). Two pigs had peak viremia at 7 dpi 10^3.75^ and 10^5.25^ TCID_50_/mL.

Samples were collected from 13 tissues including tonsil, thymus, lungs, liver, kidney, spleen, distal ileum, and six different lymph nodes. The levels of ASFV genome copies/g of tissues were generally low, although moderate levels were detected in three pigs from Group V (Table 2). Similar results were obtained in tissues from Group W pigs which were culled on 17 dpi. Moderate genome levels were detected in five tissue samples of pig W4, ranging from 10^4.2-5.0^ genome copies/g.

**Table 2.** The mean ASFV genome copies per gram tissue (in log_10_) post-immunization.

								Lymph nodes^a^					
	Ton	Thy	Lun	Liv	Kid	Spl	Ileum	SLN	RPLN	TBLN	GHLN	RLN	ICLN
Group V^b^													
V1	–	2.55	4.50	4.37	–	3.95	0.70	–	–	2.60	3.96	1.50	0.88
V2	–	0.74	2.20	2.46	–	1.54	–	2.41	3.50	5.09	3.22	–	–
V3	–	0.71	3.70	3.50	–	3.55	–	3.71	3.08	4.17	3.91	2.91	–
V4	–	2.64	2.60	0.83	–	–	1.45	2.23	2.93	4.15	2.36	4.22	–
V5	–	1.65	2.20	1.59	1.64	3.47	–	2.64	3.68	3.44	2.30	1.53	3.38
V6	0.71	–	–	–	–	–	–	–	2.18	–	–	1.42	–
Group W^c^													
W1	4.25	2.68	2.10	3.22	–	2.17	–	3.00	2.39	2.31	–	–	1.73
W2	–	–	–	3.39	–	–	–	–	–	4.15	–	–	–
W3	–	–	–	–	–	–	–	–	–	–	–	–	–
W4	5.00	4.32	4.30	2.59	–	4.22	3.48	2.87	3.22	4.68	3.68	3.95	3.60
W5	2.25	–	3.20	2.19	2.32	2.68	0.88	2.06	3.98	4.76	3.79	3.28	2.79
W6	–	1.91	–	–	2.17	–	–	–	–	–	–	1.57	–

Ton: Tonsil; Thy: Thymus; Lun: Lungs; Liv: Liver; Kid: Kidney; Spl: Spleen.

^a^ Lymph node of different origins including SLN: submandibular; RPLN: retropharyngeal; TBLN: tracheobronchial; GHLN: gastrohepatic; RLN: renal; ICLN: ileocaecal. Samples were extracted in duplicates and qPCR were performed in duplicates – quadruplicates/ sample. –: not detected.

^b^ 3 Group V pigs were culled at 7 dpi and the other 3 at 14 dpi.

^c^ Group W pigs were immunized at a high dose (10^6^) and were culled at 17 dpi.

### Immune responses of immunized pigs from experiments 1, 2, and 3

#### Antibody responses

**Figure 6.**
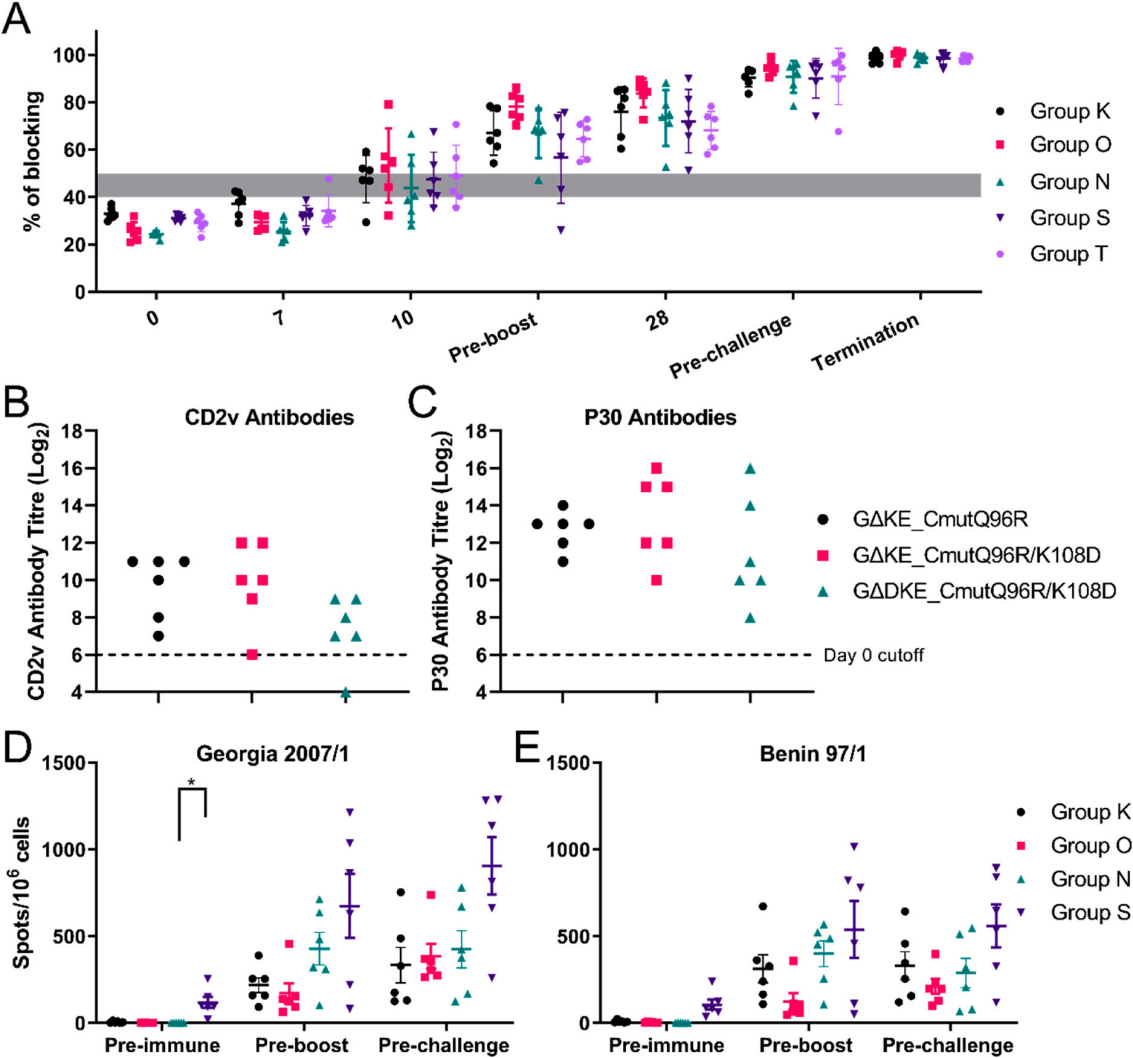
Immune responses of immunized pigs. (A) Antibody responses are shown against ASFV VP72 protein using a blocking ELISA, of pigs immunized with GΔKE_CmutQ96R (Group K), GΔKE_CmutQ96R/K108D (Group O), and GΔDKE_CmutQ96R/K108D (Groups N, S, and T) on different days after immunization and challenge (*x*-axis). Results are presented as % blocking, where values >50% blocking are positive. (B) Levels of CD2v antibody in the serum of immunized pigs, pre-challenge (Groups K, O, and N) detected by ELISA are shown. (C) Shows antibody levels against ASFV P30 protein measured by ELISA in the same pigs as in Panel B. The numbers of IFN-γ producing cells detected in PBMCs of immunized pigs (Groups K, O, N, and S) were measured via ELISpot assays. PBMCs were stimulated with either (D) Georgia 2007/1 isolate or (E) a genotype I, Benin 97/1 isolate. Results are presented as mean number of IFN-γ producing cells per million PBMC of individual pigs.

All three recombinant viruses induced ASFV P72 antibody responses as early as 10 dpi in 13 pigs and all were positive by 28 dpi (Figure 6(A)). Pigs immunized with all three viruses (Groups K, O, N) had high levels of antibodies against CD2v protein except for one pig immunized with GΔDKE_CmutQ96R/K108D (Figure 6(B)). By comparison responses to p30 antigen were higher (Figure 6(C)).

#### Interferon-γ ELIspot responses

Cellular immune responses to the recombinant viruses were measured by IFN-γ ELIspot assays before immunization, boost and challenge. Cells were stimulated with parental Georgia07/1 (Figure 6(D)). Numbers of IFN-γ producing cells increased after immunization in all pigs before boost except pig S3 (immunized with GΔDKE_CmutQ96R/K108D). However, pig S3 had increased numbers of IFN-γ secreting cells after boost. Overall, the numbers of IFN-γ secreting cells were quite variable among individual pigs in the same group. This variability was especially noticeable in pigs immunized with GΔDKE_CmutQ96R/K108D (Groups N and S) (Figure 6(D)). Stimulation of these PBMC with a genotype I, Benin97/1, isolate induced IFN-γ producing cells which increased in numbers before challenge in all pigs albeit in slightly lower levels (Figure 6(E)) suggesting cross-protection against genotype I may be induced.

## Discussion

The complexity of ASFV has hindered vaccine development. Currently, gene-deleted modified live vaccines are the most promising candidates for ASF. Several members of five different multigene families (MGF), located close to genome termini, inhibit innate immune responses, and their deletion reduces virulence. Deletion of MGF360 and MGF505 has generated candidate vaccines [2,3]. However, genome rearrangements, including deletions and transpositions between genome ends, are most commonly observed in these regions in both ASFV field isolates [21] and during passage in pigs [22]. In contrast, genome rearrangements are infrequently observed in the central genome region which codes for single copy genes [1]. Here we deleted or modified three or four single copy genes from the conserved central region of the genome, as an alternative route to construct safer live vaccine candidates. We successfully constructed recombinant viruses which induced strong early protective immune responses with minimal levels of virus replication. Opportunities for virus genome rearrangements, recombination, and transmission from immunized to naïve pigs are thus reduced.

Deletion of the EP402R gene coding for CD2v from ASFV isolates can further attenuate the virus or not depending on the genotype or virulence of the parental strain [6,23,24]. CD2v protein has been indicated to be important for protection since (i) immunization with recombinant CD2v induced partial protection [25], (ii) the ability of sera to inhibit HAD of RBC to infected cells correlated with cross-protective ability between different serogroups [26], and (iii) CD2v has both T- and B-cell epitopes [27]. Therefore, instead of deleting CD2v, we replaced wildtype CD2v gene of Georgia07/1 with a mutant form of CD2v exhibiting a partially or completely abrogated HAD phenotype. Drastic amino acid changes were introduced on the predicted ligand-binding GFCC'C″ β-sheet on genotype II Georgia07/1 CD2v protein [20] taking account of results from mutational analysis of CD2v genotype I Benin97/1 isolate (Reis et al., in preparation). Compared with human CD2, which uses unusually large numbers of charged residues to engage its ligand, CD58 [28], our model implies that CD2v is far more reliant on polar and hydrophobic contacts involving the GFCC'C″ β-sheet (Figure S2B). The alignment of protein sequences of the CD2v Ig1-domain with human and porcine CD2 (Figure 2(A)) indicates that the sequence of CD2v is very different from the animal sequences, although the overall domain structure is probably conserved. It is therefore likely that CD2v binds to different ligand(s). CD2v may bind to other cell types in addition to red blood cells, potentially explaining its immunomodulatory effects.

The first recombinant virus tested, GΔKE-CmutQ96R, expressing CD2v with a single mutation, and deletions of the EP153R and K145R genes. This virus induced few disease signs and 100% protection with almost sterile immunity after challenge with Georgia07/1. Nevertheless, these pigs had mild to moderate viremia post-immunization, and the infected cells from the blood titration pre-challenge still formed rosettes. Thus, although sterile immunity was achieved, the results suggested that this virus may not reach required safety standards.

We next tested recombinant viruses expressing CD2v with double amino acid mutations, Q96R and K108D, to abrogate HAD. The EP153R and K145R genes were also deleted allowing a direct comparison to be made of the effect of expression of CD2v that induced partial HAD with one which failed to induce HAD. DP148R was deleted from one of these strains to test the effect of this additional gene deletion. As predicted both of these strains induced lower levels of viremia compared to GΔKE-CmutQ96R. Both viruses induced protection against challenge in five of six pigs. However, in the group immunized with the virus GΔDKE-CmutQ96R/K108D, from which the DP148R gene was deleted, pigs that survived challenge showed fewer clinical signs of disease. Additional experiments with GΔDKE-CmutQ96R/K108D tested immunization with higher doses and measured tissue dissemination. These experiments confirmed that immunization with a 10-fold increased dose (Group T) induced mild clinical signs and 100% protection and low levels of virus in blood. Immunization with a 100-fold increased dose, 10^6^ TCID_50_/mL, resulted in transient mild clinical signs for a single day, except one pig that had increased body temperatures for 3 days and had moderate levels (10^5.25^ TCID_50_/mL) of virus in blood at 7 dpi. Finally, we evaluated the dissemination of GΔDKE-CmutQ96R/K108D in tissues following immunization at different doses. After immunization with 5 × 10^4^ TCID_50_/mL, ASFV DNA was detected at low levels below 10^4^ TCID_50_ in most animals in 13 tissues collected on 7 or 14 dpi. Similar results were obtained for pigs immunized with 10^6^ TCID_50_/mL (Group W) that were culled 17 dpi.

ASFV-specific CD8^+^ cellular responses are required for protection against challenge with ASFV [29]. Passive transfer of antisera from immune to naïve pigs was shown to confer partial protection indicating antibodies also have a role in protection, although the mechanisms are not clear. We observed strong early antibody and cellular immune responses in pigs immunized with all three recombinant viruses tested. High levels of ASFV-specific P72 antibodies were detected by 10 dpi in many immunized pigs and in all by 28 dpi. Levels of ASFV-specific IFN-γ secreting cells increased in immunized pigs by 21 dpi and continued to increase or maintained a plateau until challenge. In contrast, our studies with other gene-deleted attenuated ASFV strains showed that those strains that induce low viremia and clinical signs after immunization, for example with virus from which EP402R and EP153R genes were deleted, induced much lower and later immune responses and reduced protection [7,18].

Overall, we showed that GΔDKE-CmutQ96R/K108D has a safety and efficacy profile suitable for further assessment, since no or very mild, transient clinical signs, and minimal viral replication were induced after immunization. This virus could also be further modified as needed for example to delete alternative genes to achieve a good level of attenuation or other negative serology markers for DIVA assays.

## Supplementary Material

Supplemental Material
