# Supplementary material for: A protective multiple gene-deleted African swine fever virus genotype II, Georgia 2007/1, expressing a modified non-haemadsorbing CD2v protein
Source: Emerg Microbes Infect. 2023 Oct 19;12(2):2265661. doi: 10.1080/22221751.2023.2265661 (PMC10588529; doi:10.1080/22221751.2023.2265661)
Supplement: Supplemental Material [file TEMI_A_2265661_SM2291.pdf]

Supplementary data

A protective multiple gene-deleted African swine fever virus genotype II, Georgia 2007/1, expressing a modified non-haemadsorbing CD2v protein

Anusyah Rathakrishnan[1], Ana Luisa Reis[1], Vlad Petrovan[1], Lynnette Goatley[1], Katy Moffat[1], Yuan Lui[2], Mai Tuyet Vuong[2], Shinji Ikemizu[3], Simon Davis[2] and Linda Dixon[1]

Supplementary Figures

A

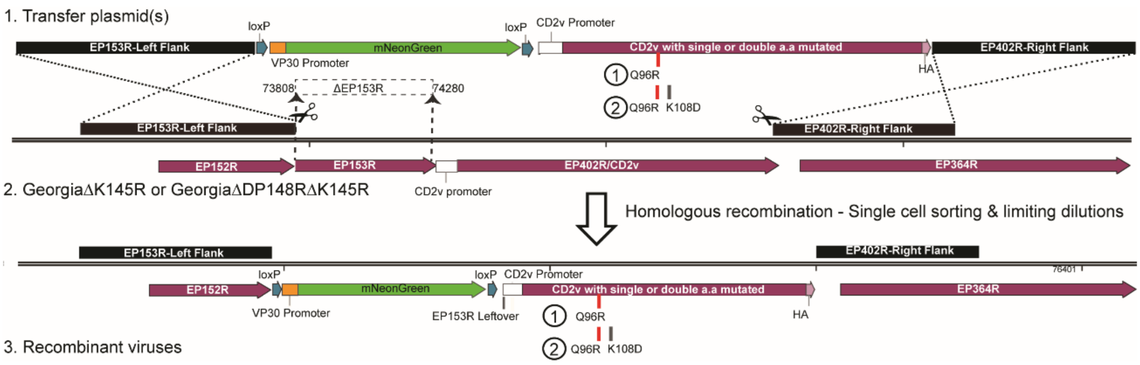

B

	Total Reads	Total Assembled Reads[a]	% Assembled Reads	Median coverage depth
GΔKE_CmutQ96R	4221420	3957490	93.7	5686.5
GΔKE_CmutQ96R/K108D	3256584	2920091	89.7	4023.3
GΔDKE_CmutQ96R/K108D	2647416	2341494	88.4	3075.2

[a] Reads were assembled under the variant analysis module (SeqMan NGen, DNASTAR) using the ASFV Georgia 2007/1 (NCBI accession number: FR682468.2) as reference guide.

Figure S1. Scheme for construction of recombinant ASFV viruses (**A**) To delete ASFV EP153R gene in combination with replacing wildtype EP402R with mutant versions, sequences from ASFV Georgia 2007/1 (FR682468.2) genome were commercially synthesised and cloned in plasmids - pΔEP153R-PP30mNG-CD2v_mutQ96R (single amino acid mutation) and pΔEP153R-PP30mNG-CD2v_mutQ96R/K108D (double amino acid mutation). These plasmids contain 720bp from the left flanking region of EP153R (position 73086 – 73805), the first LoxP site, a fluorescent reporter gene (mNeonGreen), controlled by ASFV VP30 promoter, the second LoxP site, followed by mutant versions of the EP402R gene expressing CD2v protein with an HA epitope tag fused at the 3' end. The CD2v mutations included either a single amino acid mutation at position 96 (Q → R) (position 74639 - 74641, nucleotides CAA to CgA) or double amino acid mutations at position 96 (Q → R) (position 74639 - 74641, nucleotides CAA to Cgc) and at position 108 (K → D) (position 74675 - 74677, nucleotides AAA to gAt). The CD2v protein was under control of CD2v promoter (position 74281 – 74353). Finally, a 609bp sequence flanked the stop codon at the right end of EP402R (position 75417 – 76025). The LoxP sites is to facilitate removal of the reporter gene in the future. Purified PBMs were first infected with GeorgiaΔK145R or GeorgiaΔDP148RΔK145R, recombinant ASFV lacking gene(s) K145R and/or DP148R, then transfected with the transfer plasmid from. The parental viruses both contained a red fluorescent protein, TagRFP-T. After 36–48 post infection-transfection, recombinants expressing TagRFP-T^{+} ^{and} mNeonGreen^{+}, were isolated by single cell sorting into PBMs

using FACS. Following subsequent rounds of single cell isolation and limiting dilutions, three recombinant ASFV: (i) GeorgiaΔK145RΔEP153R-CD2v_mutQ96R (GΔKE_CmutQ96R), (ii) GeorgiaΔK145RΔEP153R-CD2v_mutQ96R/K108D (GΔKE_CmutQ96R/K108D) and (iii) GeorgiaΔDP148RΔK145RΔEP153R-CD2v_mutQ96R/K108D (GΔDKE_CmutQ96R/K108D) were obtained. (**B**) Viral DNA was extracted from 3 recombinant viruses and whole genomes were sequenced. Sequences obtained were assembled using the NGS-based whole genome variant analysis module on the SeqMan NGen. The table summarizes the total number of reads, total and percentages of assembled reads and median coverage depth for each of the three recombinant viruses.

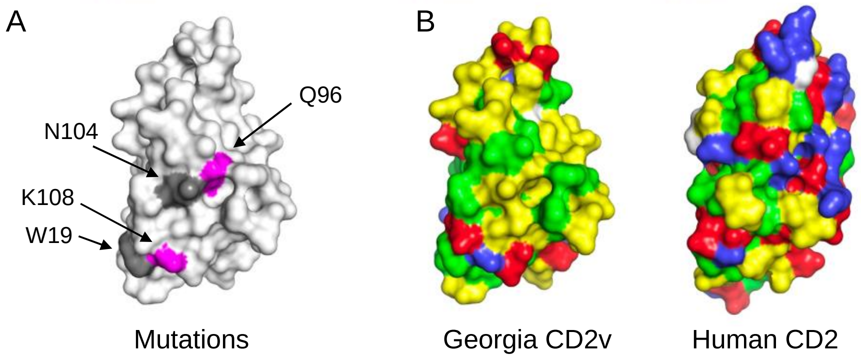

Figure S2. Surface representations of the CD2v and hCD2 immunoglobulin superfamily V-set domains. In Panel (**A**), mutations of CD2v that produced strong effects in the mutational screen are coloured magenta, and weak grey. In panel (**B**) the putative V-set domain of CD2v and that of human CD2, with the GFCC'C" β-sheets to the front, are coloured according to amino acid properties: negatively charged (D, E) – red, positively charged (R, H, K) – blue, polar (S, T, N, Q) – yellow, and hydrophobic (A, V, I, L, M, F, Y, W) – green.

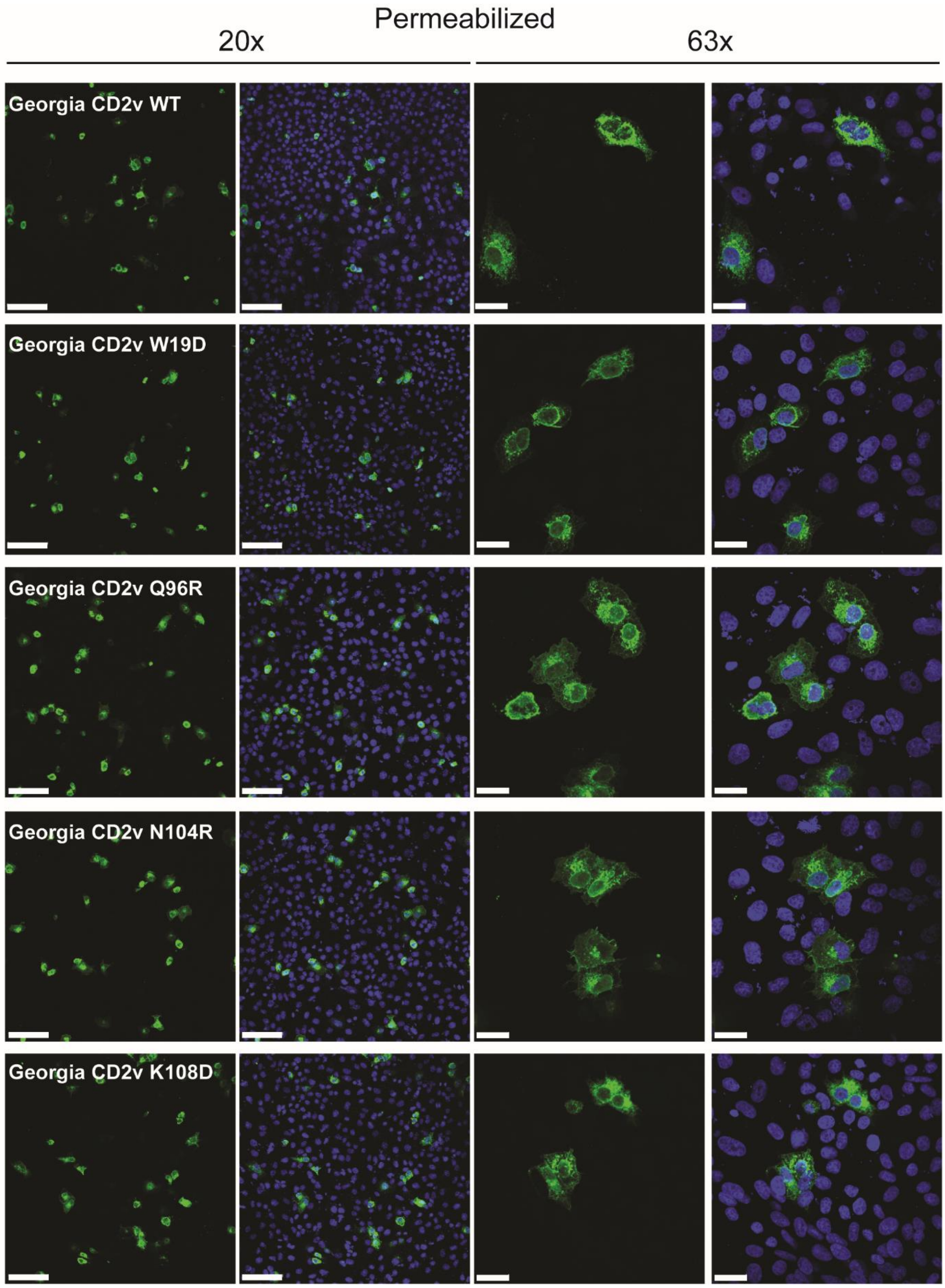

Figure S3 continues…

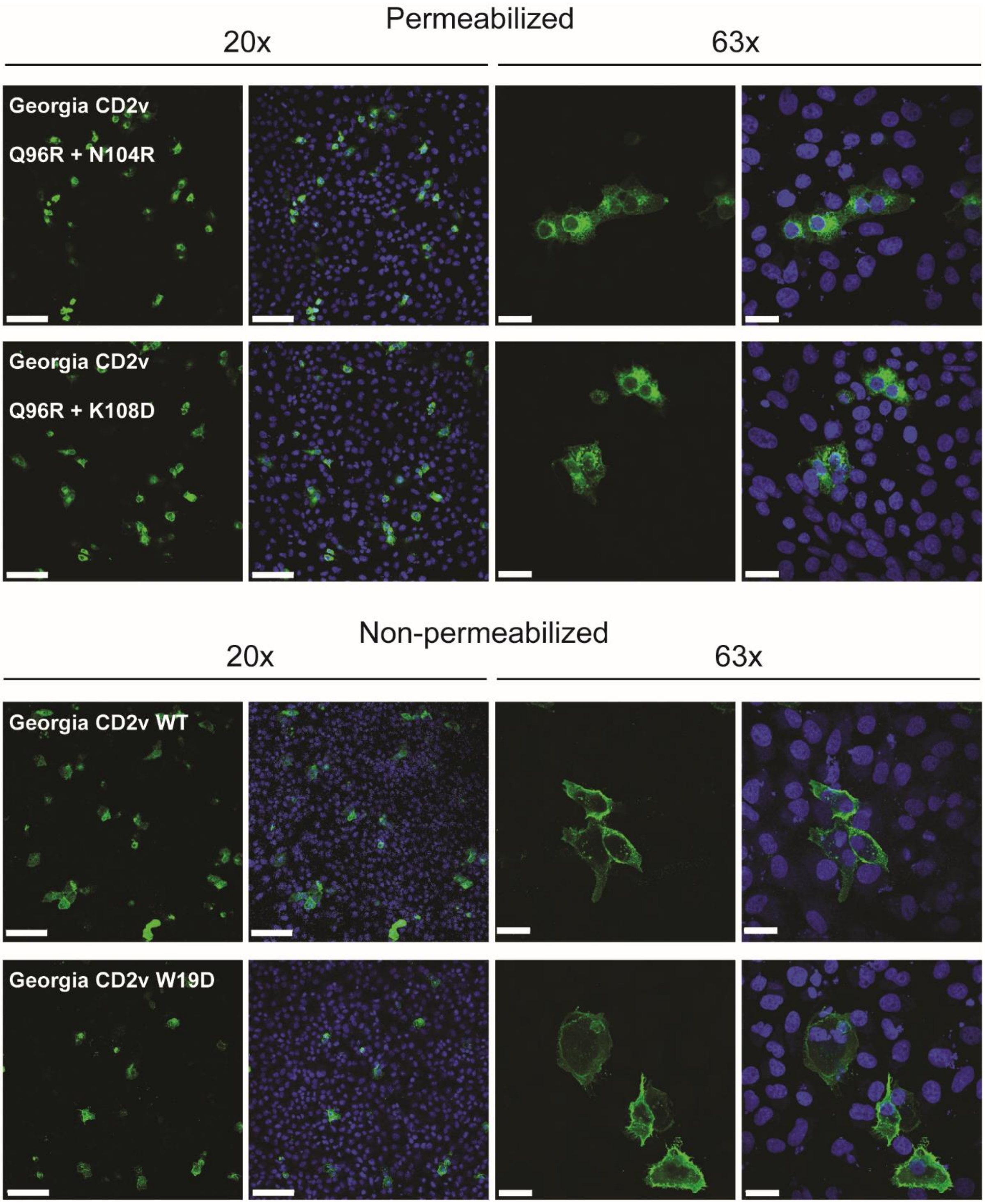

Figure S3 continues…

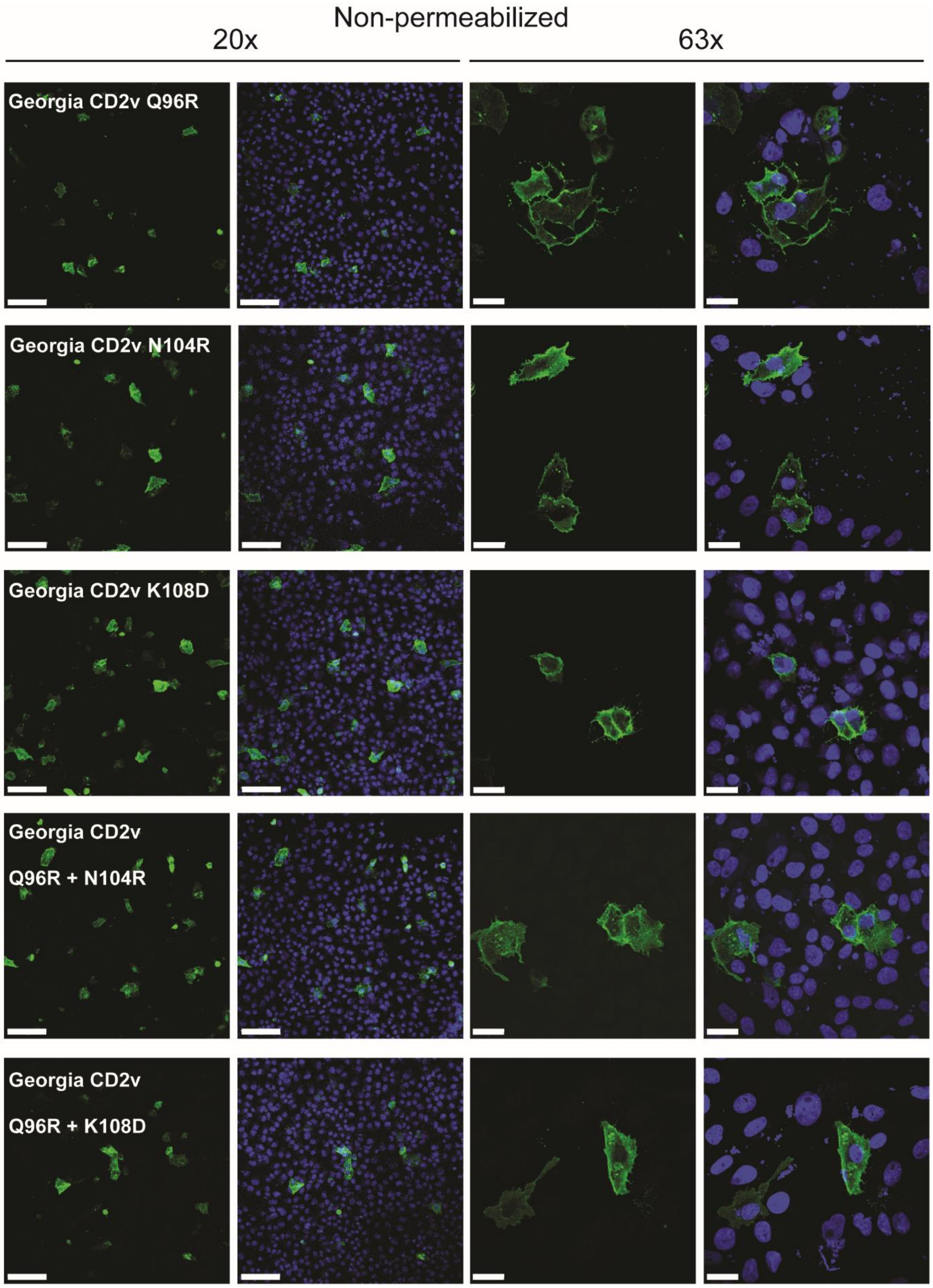

Figure S3. Transient expression of CD2v. Plasmids expressing codon-optimized Georgia CD2v, wildtype or mutants, were transfected into Vero cells. After 48h, the cells were fixed.

Coverslips were either permeabilized or non-permeabilized and probed with rat anti-HA, to detect the N-terminal HA tag fused after the signal peptide, followed by goat anti-rat Alexa-Fluor 488 and counterstained with DAPI to show the nucleus (blue). Individual panel shows the expression of CD2v (green), wildtype or mutants, inside cells (permeabilized) or on the cell surface (non-permeabilized). These are shown at magnification of 20x (scale bar represents 100µm) and 63x (scale bar represents 25µm).

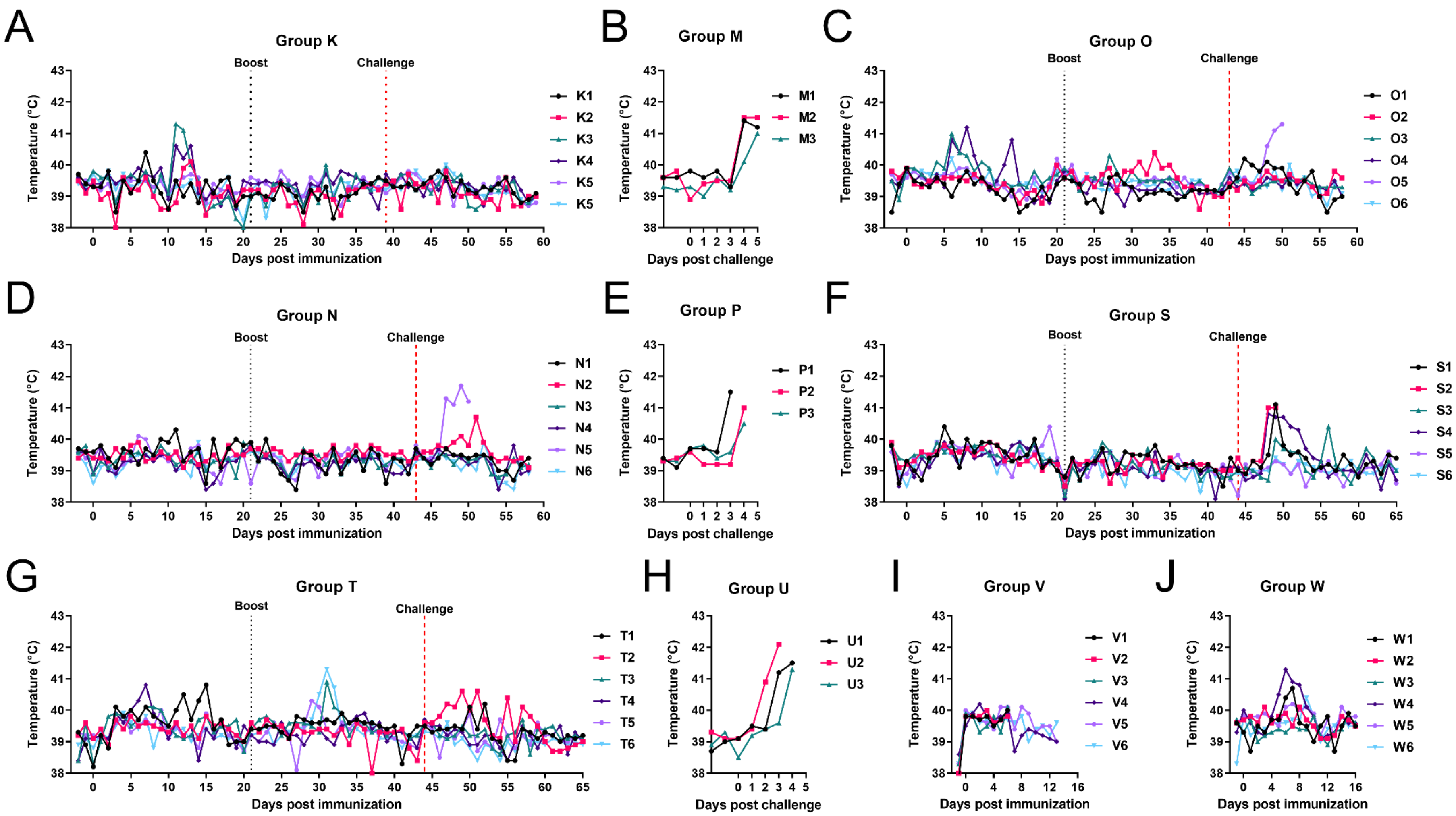
A
Group K
Boost
Challenge
Temperature (°C)
Days post immunization
K1
K2
K3
K4
K5
K5
B
Group M
Days post challenge
M1
M2
M3
C
Group O
O1
O2
O3
O4
O5
O6
D
Group N
N1
N2
N3
N4
N5
N6
E
Group P
P1
P2
P3
F
Group S
S1
S2
S3
S4
S5
S6
G
Group T
T1
T2
T3
T4
T5
T6
H
Group U
U1
U2
U3
I
Group V
V1
V2
V3
V4
V5
V6
J
Group W
W1
W2
W3
W4
W5
W6

Figure S4. Daily rectal temperatures of pigs. Rectal temperatures were recorded daily for pigs immunized with (**A**) GΔKE_CmutQ96R (Group K), (**C**) GΔKE_CmutQ96R/K108D (Group O), and (**D, F, G, I and J**) GΔDKE_CmutQ96R/K108D (Groups N, S, T, V and W). (**B, E, H**) The temperatures for the naïve, challenge control groups for each experiment were recorded following challenge with Georgia 2007/1 isolate.

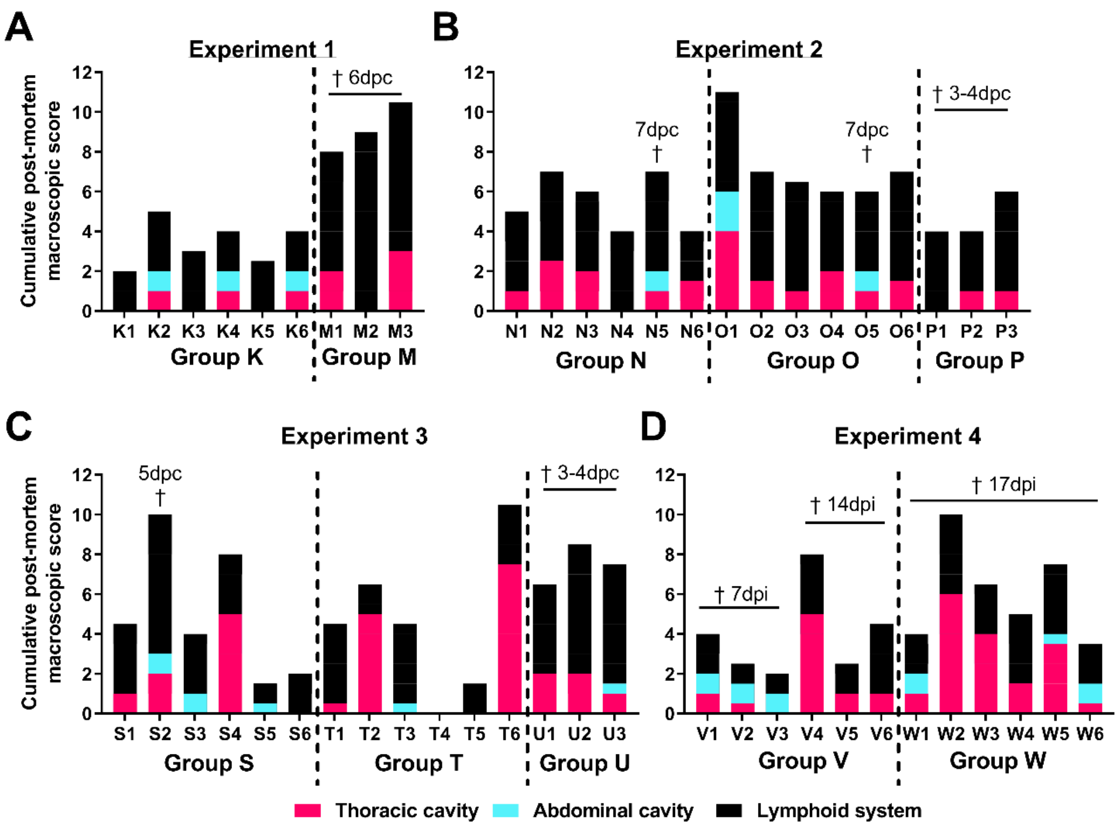

Figure S5. Post-mortem macroscopic lesion scoring. Lesions observed during post-mortems were scored and displayed as a cumulative score observed in different sections of the pig. Results for individual pigs in experiments 1 to 4 are shown in panels **A** to **D**. Lesions in the thoracic cavity (pink) include the presence of thoracic exudates as well as lesions affecting cardio-respiratory system. Lesions in the abdominal cavity (blue) include the presence of ascites along with the presence of lesions affecting the gastrointestinal system including the stomach, intestines, liver, and gallbladder. Lesions in the lymphoid tissues (black) include the tonsils, thymus, spleen, and various lymph nodes. The † above the individual or grouped bars denotes pig(s) that were culled before the end of the experiments and the day of cull for experiments 1 – 3. In experiment 4, 3 Group V pigs were culled at 7dpi and the other 3, at 14dpi, while Group W pigs were all culled 17dpi at the end of the experiment.

Table S1: The mean ASFV genome copies per gram tissue (in $\log_{10}$) post- challenge.

	Ton	Lun	Spl	SLN	GHLN	RLN
**Group S**						
S1	3.51	2.10	2.16	1.37	-	2.58
S2†	1.45	3.90	6.86	0.69	2.32	1.47
S3	3.57	2.10	4.02	1.46	4.32	1.47
S4	3.74	3.80	3.57	-	0.79	-
S5	-	-	-	1.64	-	2.90
S6	-	2.20	-	-	-	2.87
**Group T**						
T1	-	1.60	-	-	1.41	0.77
T2	3.92	3.30	2.82	-	3.43	3.53
T3	2.70	2.30	0.78	-	-	0.76
T4	-	-	-	0.74	-	-
T5	-	-	-	-	-	-
T6	-	-	-	-	-	-
**Group U**						
U1†	5.03	6.4	6.74	4.908	4.19	4.77
U2†	5.55	7.3	8.31	5.813	6.08	5.68
U3†	5.42	7.2	7.85	5.773	5.86	6.18

Ton: Tonsil; Thy: Thymus; Lun: Lungs; Liv: Liver; Kid: Kidney; Spl: Spleen; SLN: submandibular lymph nodes; RPLN: retropharyngeal lymph nodes; TBLN: tracheobronchial lymph nodes; GHLN: gastrohepatic lymph nodes; RLN: renal lymph nodes; ICLN: ileocaecal lymph nodes. Samples were extracted in duplicates a- qPCR were performed in duplicates – quadruplicates/ sample. - : not detected. † Pigs culled at moderate endpoints.

Supplementary Methods

Purification of CD2v protein

Recombinant protein of the extracellular domain of CD2v from ASFV genotype IX containing a C-terminal C-Tag (EPEA) was stably expressed in HEK293T cells as a secreted form via lentiviral transduction. The protein was affinity purified by binding to CaptureSelect C-tagXL beads (Thermo Scientific), washing with PBS pH7.4, then eluted using 3mM C-Tag peptide (SEPEA, Genscript) in PBS. Fractions above A280 of 0.1 were collected, concentrated to 0.5mL with Amicon Ultra-15 10KDa concentrator (Millipore), and SEC on Superdex 75 10/300 GL column (GE, now Cytiva) connected to AKTA Pure (GE) into PBS.

Immunofluorescence staining of CD2v

Vero cells on coverslips, transiently expressing wildtype of mutant CD2v were fixed with 4% paraformaldehyde 48 h post-transfection. Cells were permeabilized with 0.2% Triton X-100 (Sigma, UK) to detect intracellular localization of CD2v. A duplicate coverslip of the same transfection was not permeabilized to detect expression of CD2v on the cell surface. After blocking with 0.5% bovine serum albumin (BSA) in PBS-T (0.1% Tween-20 in PBS), cells were incubated with rat anti-HA (Sigma, UK). The HA tag was fused to the N-terminal of CD2v, after the signal peptide. After the addition of goat anti-rat IgG (H+L) cross-adsorbed Alexa Fluor™ 488 secondary antibodies, nuclei were stained with DAPI. Finally, coverslips were mounted of glass slides containing Vectashield antifade mounting medium. The coverslips were sealed and imaged under the Leica SP8 CLSM, upright microscope. The number of cells expressing CD2v were quantified using Imaris software (Oxford Instruments). One-way ANOVA with Tukey's multiple comparison test was performed to evaluate the differences in expression levels of wildtype and mutant CD2v intracellularly and

on the surface.

Production of ASFV recombinant Georgia07/1 with deletions of K145R, EP153R only or with DP148R and expressing modified CD2v proteins

To delete the EP153R gene combined with replacing wildtype EP402R with mutant versions by homologous recombination, sequences from the ASFV Georgia 2007/1 (FR682468.2) genome were commercially synthesised and cloned in plasmids (Genscript US). These transfer plasmids, pΔEP153R-PP30mNG-CD2v_mutQ96R and pΔEP153R-PP30mNG-CD2v_mutQ96R/K108D, contain 720bp sequences upstream of EP153R, mNeonGreen reporter controlled by P30 promoter, LoxP sites flanking the mNeonGreen marker, mutant versions of EP402R with an HA epitope tag fused at the 3' end, and 609bp sequence downstream the stop codon of EP402R (Figure S2A). The mutant EP402R contained either a single amino acid mutation at position 96 (Q → R) (FR682468.2, position 74639 - 74641, nucleotides CAA to CgA) or double amino acid mutations at position 96 (Q → R) (FR682468.2, position 74639 - 74641, nucleotides CAA to Cgc) and at position 108 (K → D) (FR682468.2p, position 74675 - 74677, nucleotides AAA to gAt).

Recombinant viruses expressing both TagRFP-T and mNeonGreen were isolated via a combination of single cell FACS sorting and rounds of limiting dilutions [1]. Full genome sequencing of recombinant ASFV using an Illumina MiSeq instrument using a 600 cycle v3 cartridge. Sequences obtained were assembled using the NGS-based whole genome variant analysis module on the SeqMan NGen (DNASTAR Lasergene).

Multistep growth curves

Purified PBMs from 2 different pigs were infected at a multiplicity (MOI) of 0.01 in triplicates. Total infectious virus titres were determined and repeated measure two-way

ANOVA, with Dunnett's multiple comparisons test was performed to compare replication of recombinant and wildtype ASFV.

Western blots

Lysates from Vero cells transiently expressing wildtype or mutant CD2v or purified PBMs infected with recombinant ASFV were separated by SDS/PAGE and transferred onto Amersham Hybond P 0.45 PVDF blotting membranes (Cytiva). Blots were probed with rat anti-HA monoclonal antibody (Roche) and secondary goat anti-rat IgG HRP conjugated antibody (Invitrogen). Bound antibodies were detected by ECL Select (Amersham) and imaged on Syngene G: Box.

In vivo immunization and challenge

Animal experiments were carried out at the Pirbright Institute under UK Home Office License 7088520. Groups of 6 female Landrace- Large White cross pigs with initial weights of 17 - 30kg were inoculated and challenged via the intramuscular route (IM). Group of 3 non-immune pigs were challenged in parallel to act as controls. Clinical signs were recorded daily and blood samples were collected on selected days [2].

Figure 1 shows the schedule of 4 experiments conducted. In experiment 1, one group (K) was immunised with GeorgiaΔKE-CmutQ96R. In experiment 2, one group was immunised with GeorgiaΔKE-CmutQ96R/K108D (O) and a second group with GeorgiaΔDKE-CmutQ96R/K108D (N). In experiment 3 two groups of pigs were immunised with different doses of GeorgiaΔDKE-CmutQ96R/K108D. In experiment 4, 2 groups of pigs were immunized with two higher doses GeorgiaΔDKE-CmutQ96R/K108D and culled between 7 - 17 days to investigate the tissue dissemination of the virus. Viruses were back titrated to verify dosages used.

Pigs were euthanized at termination of the experiment or at a moderate severity humane endpoint, typically following 4 days of temperature ≥40.6°C or 3 days of temperature combined with other clinical signs. Macroscopic lesions associated with ASFV were scored [3].

Viremia measurement

Whole blood was collected in EDTA from pigs at different days after immunization and challenge. Blood was serially diluted and titrated in PBMs by HAD_{50}/mL or $TCID_{50}$/mL on PBMs in quadruplicate.

Nucleic acid isolation from pig tissues

Tissues collected during post-mortem were stored at -80ºC. The tissues were excised and added to Lysing Matrix A tubes (MP Biomedicals™) containing 500µL PBS and supplemented with 1% pen-strep. The tissues were then homogenized on a BeadBug™ microtube homogenizer (Benchmark Scientific) for 3 minutes at maximum speed. DNA was extracted in duplicates from 20mg of homogenized tissue using the MagMAX™ CORE Nucleic Acid Purification Kit (Thermo Fisher Scientific) on a Kingfisher Flex Extraction System, with the MagMAX_Core_Flex_NO_Heat script.

ASFV genome detection in tissues

ASFV DNA was detected Extracted DNA from tissues were assayed in duplicates for the presence of viral DNA by qPCR on an AriaMx real-time PCR system (Agilent) following a protocol modified using the primers by qPCR using and probes primers against ASFV VP72 [4]. A standard curve was prepared from a p72 mimic plasmid by making serial dilution ranging from 10 to 10^8 copies/mL. Results were reported as log10 genome copies/mL. A cut-

off for accurate detection was based on the serial 10-fold dilutions of a control plasmid, where the lowest value is 10 copies. This translates to a cut-off accurate measurement of 5 x 10^3 ASFV genome copies/mL. Below this, the exact genome copy cannot be determined. We defined levels of virus genome less than 10^4 genome copies/mL as low, 10^4 to 10^6 genome copies/mL as moderate and above 10^6 as high.

Detection of ASFV p72 antibody responses in pigs

Pig serum was extracted by centrifugation from whole blood collected in BD serum vacutainers, at various days post immunization and challenge. The antibody responses against ASFV-p72 were measured using a commercial blocking ELISA (INgezim PPA Compac, Ingenasa) following the manufacturer's instructions. The optical densities (OD) were read at 450nm on a microplate reader BioTek with Gen5 software. The percentage of blocking was calculated using the following formula: [(negative-control OD − sample OD) / (negative-control OD − positive-control OD)] × 100, where OD is optical density. Samples above 50% blocking were considered as positive, 40 to 50% as doubtful and while anything below 40% was considered as negative. Samples with blocking between 40-50% are considered as doubtful.

Detection of ASFV CD2v and P30 antibody responses in pigs

Recombinant CD2v protein was produced as described above while recombinant P30 was obtained from Native Antigen Company. Both antigens were coated at 2µg/mL on different MaxiSorp™ plates (Nunc) overnight at 4°C. Serially diluted matching pig serum (day 0 and pre-challenge) from Groups K, O and S were 2-fold serially diluted (1: 64 to 1: 65536) in blocking buffer, added to plates and incubated at 37°C for an hour. Following an hour incubation with goat anti-pig IgG (H+L) antibody, HRP conjugated (Bethyl Laboratories),

bound antibodies were detected by treatment with 1:1 tetramethylbenzidine (TMB) substrate and hydrogen peroxide (R&D Systems) at room temperature for 10 – 15 min. The reaction was stopped with 2N H_2SO_4 and absorbance were measured at 450nm on BioTek plate reader with Gen5 software. The cut-off was 1:64 based on the day 0 samples. Repeated measure two-way ANOVA, with Tukey's multiple comparisons test was performed to compare the differences between groups.

IFN-γ ELISpot assay to measure cellular responses in pigs

PBMCs collected were stimulated overnight with 200µl with 10^5 HAD_{50} of ASFV Georgia07/1 an equivalent volume of mock inoculum, or 20µg/mL phytohemagglutinin as a positive control. After lysis and incubation with biotinylated anti-porcine IFN-γ monoclonal antibody (Invitrogen) followed by streptavidin alkaline phosphatase (Caltag), the AP conjugate substrate kit (Bio-Rad) was used to develop spots. The spot forming cells (SFC) were counted using an ELISpot assay reader system (Immunospot, CTL). Repeated measure two-way ANOVA, with Tukey's multiple comparisons test was performed to compare the differences between groups at the three different time points.

Supplementary References

1. Rathakrishnan A, Reis AL, Moffat K, et al. Isolation of Porcine Bone Marrow Cells and Generation of Recombinant African Swine Fever Viruses. In: Netherton CL, editor. African Swine Fever Virus: Methods and Protocols. New York, NY: Springer US; 2022. p. 73-94.
2. King K, Chapman D, Argilaguet JM, et al. Protection of European domestic pigs from virulent African isolates of African swine fever virus by experimental immunisation. Vaccine. 2011 Jun 20;29(28):4593-4600.
3. Galindo-Cardiel I, Ballester M, Solanes D, et al. Standardization of pathological investigations in the framework of experimental ASFV infections. Virus research. 2013;173(1):180-190.

4. King DP, Reid SM, Hutchings GH, et al. Development of a TaqMan® PCR assay with internal amplification control for the detection of African swine fever virus. Journal of Virological Methods. 2003 2003/01/01/;107(1):53-61.
